# Prospect of quantum anomalous Hall and quantum spin Hall effect in doped kagome lattice Mott insulators

**DOI:** 10.1038/srep25988

**Published:** 2016-05-17

**Authors:** Daniel Guterding, Harald O. Jeschke, Roser Valentí

**Affiliations:** 1 Institut für Theoretische Physik, Goethe-Universität Frankfurt, Max-von-Laue-Straße 1, 60438 Frankfurt am Main, Germany

## Abstract

Electronic states with non-trivial topology host a number of novel phenomena with potential for revolutionizing information technology. The quantum anomalous Hall effect provides spin-polarized dissipation-free transport of electrons, while the quantum spin Hall effect in combination with superconductivity has been proposed as the basis for realizing decoherence-free quantum computing. We introduce a new strategy for realizing these effects, namely by hole and electron doping kagome lattice Mott insulators through, for instance, chemical substitution. As an example, we apply this new approach to the natural mineral herbertsmithite. We prove the feasibility of the proposed modifications by performing *ab-initio* density functional theory calculations and demonstrate the occurrence of the predicted effects using realistic models. Our results herald a new family of quantum anomalous Hall and quantum spin Hall insulators at affordable energy/temperature scales based on kagome lattices of transition metal ions.

The kagome lattice structure, which consists of corner-sharing triangles, is notorious for supporting exotic states of matter. For instance, the possible experimental realization of quantum spin-liquids based on spin-1/2 kagome lattices has generated in the past intense research efforts on herbertsmithite and similar frustrated antiferromagnets^1^,^2^,^3^,^4^,^5^,^6^,^7^,^8^,^9^,^10^,^11^,^12^. Recently, the kagome lattice has also received plenty of attention for quasiparticle excitations with non-trivial topology^13^,^14^,^15^. From topologically non-trivial electronic bands, effects such as the quantum spin Hall effect (QSHE)^16^,^17^,^18^ and the quantum anomalous Hall effect (QAHE)^19^,^20^ can emerge, also in kagome lattices^21^,^22^. A quantum spin Hall insulator in two dimensions, also known as a topological insulator, is a topological state of matter, present in a system with spin-orbit coupling, where symmetry protected dissipationless spin-polarized currents counterpropagate on the sample edges, while the bulk of the sample remains insulating (Fig. 1a). This phenomenon has received considerable attention because Majorana bound states have been predicted to appear at interfaces between QSHE materials and superconductors^18^,^23^,^24^. Employing these Majorana zero modes for topological quantum computation is a rapidly developing field^25^.

In contrast to the QSHE, in a quantum anomalous Hall insulator only one spin species propagates around the sample edge due to the presence of intrinsic magnetization in the sample (Fig. 1b). This state of matter offers a direct realization of *intrinsic* topological properties in a material through the combination of spin-orbit coupling and magnetism^17^. Due to the dissipation-free, spin-polarized edge currents in the absence of external magnetic fields, realizations of the QAHE are also intensively sought for, especially for application in new energy-efficient spintronic devices^26^,^27^. So far, in electronic systems the QAHE has only been observed in thin films of chromium-doped (Bi, Sb)_2_Te_3_ at a temperature of 30 mK^28^,^29^, the main limitation being the low Curie temperature of the material involved. Lately, it has been proposed that the QAHE can be realized in some other compounds using, for instance, manipulated surfaces or exfoliated monolayers^30^,^31^,^32^. Another interesting approach is design from scratch of organometallic networks with topological bandstructures^33^,^34^. A good strategy for designing QAHE compounds based on existing materials with favorable energy scales that are adequate for applications is however currently lacking.

Here, we propose a new approach to create materials with non-trivial band topology and large Curie temperatures, exploiting the electronic properties of doped Mott insulators on a kagome lattice. A quick look at the one-electron properties (bandstructure) of the kagome lattice with nearest neighbor hoppings (Fig. 2) shows huge potential for the realization of possible exotic states by only varying the electron filling. At half-filling the Fermi level lies near a van Hove singularity and inclusion of many-body correlation effects renders the system a Mott insulator^35^. At a filling of *n* = 4/3, however, the Mott transition is absent^12^ and the Fermi level is at the Dirac point, where non-trivial band effects may be expected upon consideration of spin-orbit coupling. The spin-orbit coupling opens a gap at the position of the Dirac point and the non-trivial topology of electrons on the kagome lattice leads to surface states of both spin species that traverse the bulk band gap opened by relativistic effects and, the QSHE is realized.

Even more interesting is the filling of *n* = 2/3 with the Fermi level right at the flat band. It was recently suggested^36^ that if a nearly flat band is partially filled, a proper combination of spin-orbit coupling, ferromagnetism and geometric frustration will give rise to the fractional quantum Hall effect at high temperatures. Along these lines, we exploit here as a key ingredient for topological non-trivial states, the tendency towards ferromagnetism^37^ of a filled flat band in hole-doped transition-metal-based kagome lattices. At *n* = 2/3 the ferromagnetic instability combined with correlation effects is expected to gap out one spin-channel and move the Fermi level of the other spin-channel exactly to the Dirac point. When spin-orbit coupling (SOC) is considered, we have the same situation as for the filling of *n* = 4/3 but only for one spin species. In such a situation, the QAHE with fully spin-polarized dissipation-free surface states is realized.

To demonstrate this new strategy of finding QSHE and QAHE materials by doping Mott insulators, we investigate which possible modifications of the natural mineral herbertsmithite -a Mott insulator with spin-liquid behavior- leave the perfect kagome motif undistorted and realize different electronic fillings.

Herbertsmithite crystallizes in the centrosymmetric space group 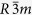 and its structure is based on layers of Cu^2+^ (3*d*^9^) ions building a perfect two-dimensional half-filled frustrated kagome lattice separated by layers of Zn^2+^ ions (Fig. 3a). The Cu atoms are in a square planar crystal field environment of oxygen ions so that the orbitals near the Fermi level are correlated 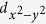 states. Evaluating density functional theory (DFT) total energies, we show that single crystals of materials obtained by following various doping choices in herbertsmithite can in principle be synthesized. Further, we prove that the magnetic ground state of hole-doped herbertsmithite at filling 2/3 is ferromagnetic, which validates that the flat band physics of the pure kagome lattice carries over to realistic situations. Finally, we demonstrate the presence of topologically non-trivial surface states of doped herbertsmithite using a state-of-the-art Wannier function technique based on fully relativistic DFT calculations.

## Materials and Methods

We prepared hypothetical materials starting from the experimental crystal structure of herbertsmithite^1^, substituting zinc (Zn^2+^) atoms between the copper kagome layers (see Fig. 3) by monovalent *A* = Li^+^, Na^+^ (hole-doping) and trivalent Al^3+^, Ga^3+^, In^3+^, Sc^3+^, Y^3+^ (electron-doping). We refer to these compounds as *A*-herbertsmithite, *A*Cu_3_(OH)_6_Cl_2_.

Experimental and hypothetical crystal structures were fully relaxed using DFT in the projector augmented wave (PAW) formulation^38^ implemented in GPAW^39^ with a plane-wave cutoff of 1000 eV and the GGA exchange-correlation functional^40^. We optimized the stoichiometric structures using 6^3^ *k*-points (4^3^ *k*-points for non-stoichiometric structures) until forces were below 10 meV/Å.

For each of the substituted structures with perfect copper kagome layer we also constructed a defect structure, where we lowered the symmetry of the unit cell and exchanged the substituent *A* with a copper atom from a kagome lattice site. As the chemical composition of these defect structures is identical to the defect-free structures, energy differences can be evaluated directly within DFT. In case the defect structure has lower energy, the kagome lattice is likely to be destroyed and the phenomena of interest here will not arise in the target compound.

Total energies, electronic bandstructures and magnetic exchange interactions of the relaxed structures were then evaluated using *ab-initio* DFT calculations within an all-electron full-potential local orbital (FPLO)^41^ basis. For the exchange-correlation functional we employed the generalized gradient approximation (GGA)^40^, as well as DFT+U^42^ functionals. The latter was necessary in order to treat the correlated nature of Cu 3*d* orbitals. The Hubbard repulsion on the Cu 3*d* orbitals was set to *U* = 6 eV and Hund’s rule coupling to *J*_*H*_ = 1 eV. Although we concentrate our investigation on the Cu 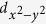 orbitals close to the Fermi level, the interactions were included in the entire Cu 3*d* shell, which is spread out over a large range of energies due to the interaction with the ligands. Additionally, we investigated the effect of spin-orbit coupling on the electronic bandstructure employing the fully relativistic version of the FPLO code. Total energies, electronic bandstructures, tight-binding and Heisenberg models were extracted from calculations converged using 8^3^, 20^3^, 40^3^ and 6^3^ *k*-point grids respectively.

To demonstrate the existence of surface states, we constructed bulk tight-binding models for the copper states (*n, j, m*_*j*_) = (3, 5/2, ±5/2) from fully relativistic DFT calculations using projective Wannier functions^43^. Employing a method based on Green’s functions^44^,^45^,^46^, we calculate the states on the surface of herbertsmithite. The spectral function is obtained from the Green’s function as 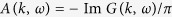.

## Results

### Stability estimates

By performing exhaustive DFT calculations we identified as the limiting factor for modifying herbertsmithite the tendency of certain ions towards substituting copper sites in the kagome layer. In Fig. 3b we plot the energy difference (tabulated in the Supplemental Information) between substitution at the kagome site and substitution at the interlayer site for herbertsmithite as a function of the substituent ionic radius^47^. In herbertsmithite, sodium (Na^+^) and yttrium (Y^3+^) prefer to occupy a site in the kagome layer, which generates a monoclinically distorted crystal structure with no perfect kagome lattice. If the substituent atom occupies the interlayer site, the perfect kagome motif is preserved.

In terms of substitution energies, lithium (Li^+^) is the most promising candidate for synthesis of hole-doped herbertsmithite. On the electron-doped side, aluminum (Al^3+^), gallium (Ga^3+^) and scandium (Sc^3+^) are the most promising candidates for substitution. Formation of the substituted materials is found to be energetically favorable compared to the formation of the parent compound clinoatacamite, Cu_2_(OH)_3_Cl. All herbertsmithite-based materials investigated are stable against formation of vacancies and copper impurities, as opposed to full substitution, on the interlayer site. We also investigated fractional substitution of Zn^2+^ by Ga^3+^ and found that the doping series Ga_*x*_Zn_1−*x*_Cu_3_(OH)_6_Cl_2_ should be stable in a broad range of Ga:Zn ratios (see Supplemental Information).

### Electronic and magnetic properties

**Figure 2:**
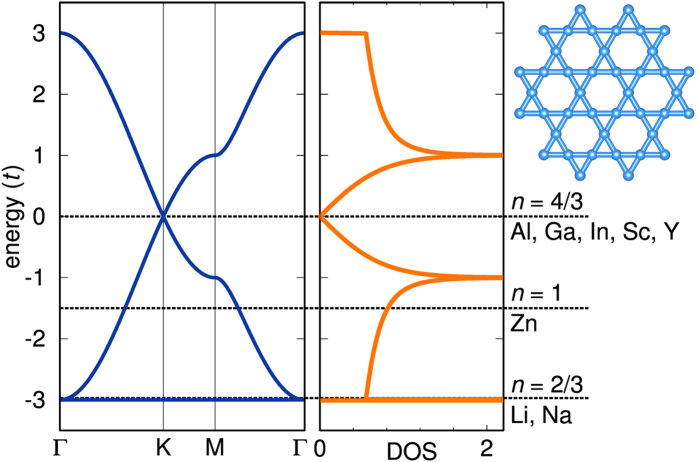
Electronic bandstructure and density of states of the pure kagome lattice (shown in upper right corner). On the right hand side the possible substituent elements for the herbertsmithite system with corresponding Fermi level are listed.

**Figure 3:**
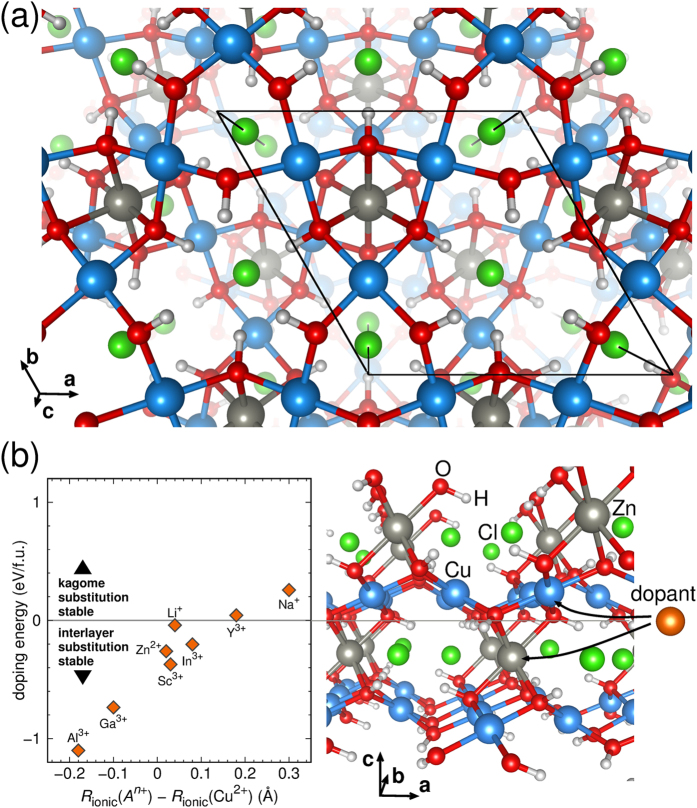
Crystal structure and calculated doping energies for herbertsmithite. (**a**) View of the crystal structure of herbertsmithite along the c-axis, which is perpendicular to the copper kagome layers. The Cu atoms (shown in light blue) are in a square planar crystal field environment of oxygen ions (red) so that the orbitals near the Fermi level are correlated 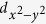 states. Each oxygen atom bonds to a hydrogen atom (white) located outside the copper and oxygen layer. In this interlayer space also zinc (grey) and chlorine (green) atoms are located. (**b**) The left hand side shows doping energies for herbertsmithite. All data points above the zero energy line indicate that the kagome lattice will be distorted upon doping. The right hand side shows the crystal structure of herbertsmithite with two possible sites for substitution indicated. On the interlayer site (Zn position, negative doping energy), herbertsmithite mostly prefers to incorporate ions with smaller radius than Cu^2+^. All ions with positive doping energy occupy the Cu position and distort the kagome lattice. Ionic radii in coordination number 6 are taken from ref. 47.

**Figure 4:**
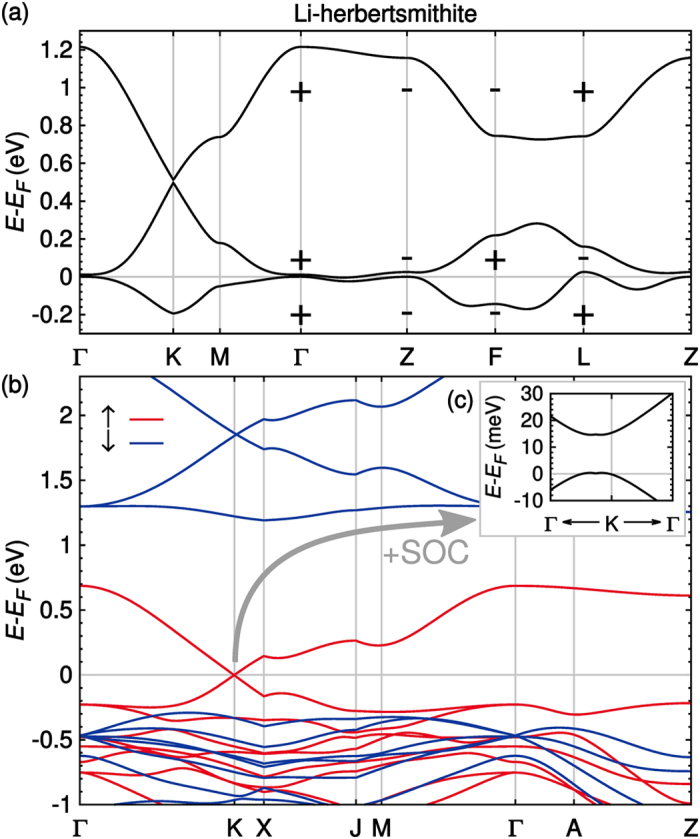
(**a**) Fully relativistic non-spin-polarized bandstructure. Plus and minus signs denote the parities of the three bands closest to the Fermi level at eight inversion-symmetric points (Γ, 3 × *F*, 3 × *L, Z*) in the Brillouin zone. (**b**) FM bandstructure without SOC. The spin down channel is gapped, while the Dirac point of the spin up channel is located exactly at the Fermi level. The path through the Brillouin zone is shown in the Supplemental Information. (**c**) Fully relativistic FM bandstructure close to the Dirac point. SOC opens a gap.

In the analysis of the electronic and magnetic properties we concentrate here on the hole-doped materials (*n* = 2/3, as defined in Fig. 2) where the Fermi level lies in the region of the flat band of the kagome lattice and a strong ferromagnetic instability is to be expected. Figure 4a displays the fully relativistic non-spin-polarized electronic bandstructure of Li-herbertsmithite, where the parities of the three (dominantly Cu 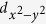) bands closest to the Fermi level are also indicated. Due to the bulk nature of the system, the ideal kagome flat band acquires some dispersion. Nonetheless, ferromagnetism is strongly favored. Indeed, total energy calculations of Li-herbertsmithite in various copper spin configurations give ferromagnetism as the ground state. Furthermore, parametrizing the Cu-Cu interactions by mapping DFT+U total energies to a spin-1/2 Heisenberg Hamiltonian shows a large ferromagnetic nearest neighbor exchange *J*_1_ = 544 K and other couplings with magnitude smaller than 0.1*J*_1_. Using a mean-field approximation^48^, we estimate a Curie temperature of *T*_*C*_ ≈ 1160 K (see Supplemental Information for further details).

Figure 4b displays the non-relativistic ferromagnetic bandstructure obtained with the DFT+U functional. The Fermi level of the majority bands lies right at the Dirac point and inclusion of spin orbit coupling opens a gap of about 15 meV (see inset in Fig. 4). The Dirac point position is slightly displaced from *K* due to the finite coupling between the kagome layers, as has been observed previously in ref. 12. The position of the Fermi level is understood by comparing the results to the spin-degenerate bandstructure shown in Fig. 2. The fully spin-polarized ferromagnetic state occurs at *n* = 2/3, therefore the spin-resolved fillings are *n*_↑_ = 2/3 and *n*_↓_ = 0. As a consequence, the bands of the majority spins resemble the non-spin-polarized case at *n* = 4/3 (where 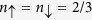) and the minority spins are empty and gapped. The ferromagnetic instability places therefore the Fermi level of the up spin bands right at the Dirac point.

To show the presence of topologically protected edge states in doped herbertsmithite, we calculated the product of parity eigenvalues at eight inversion-symmetric points (see Fig. 4) in the Brillouin zone (Γ, 3 × *F*, 3 × *L, Z*)^49^. For all materials of the herbertsmithite family, topological numbers of the bands below the Dirac point are 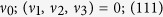. These indices indicate that the system realizes a stack of two-dimensional topological insulators (so-called *weak* TI), which displays conducting states on a (001) surface^49^, although the bulk bands are gapped by relativistic effects.

Note that non-trivial band topology is intrinsic to the perfect kagome lattice^21^,^22^ and no particular inversion of orbital weights is required unlike in most topological insulators^28^,^50^. In real materials however, the kagome layer is embedded into a crystal, where non-trivial band-topology can be destroyed by additional hybridizations. We observed this case for instance in test calculations for modifications of the natural mineral barlowite, *A*Cu_3_(OH)_6_FBr^51^,^52^, which has a crystal structure similar to herbertsmithite with perfect kagome layers.

### Demonstration of surface states

**Figure 5:**
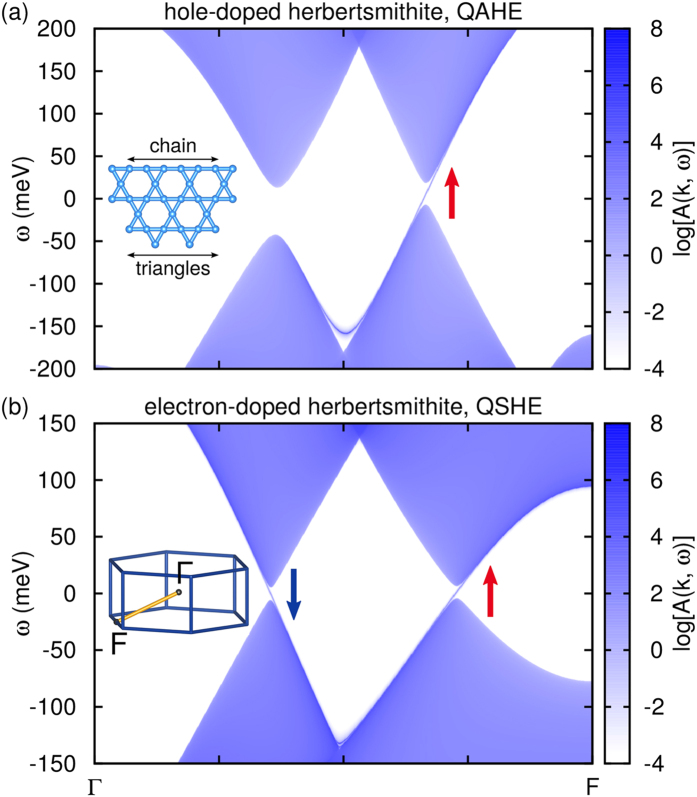
Calculated surface states of substituted herbertsmithite. Spectral function on the (001) surface of (**a**) Li-herbertsmithite (hole-doped) and (**b**) Ga-herbertsmithite (electron-doped) calculated using Green’s functions for the semi-infinite system. The arrows pointing upwards/downwards stand for the *m*_*j*_ = +5/2 and −5/2 states respectively. The inset of (**a**) shows the two possible terminations of the kagome lattice. The inset of (**b**) shows the path in the hexagonal Brillouin zone.

Having found non-trivial band-topology in the herbertsmithite system, we predict that hole-doped herbertsmithite shows a QAHE at *n* = 2/3 filling while electron-doped herbertsmithite shows a QSHE at *n* = 4/3 filling. For both cases we constructed realistic tight-binding models for the orbitals close to the Fermi energy and calculated the states on the (001) plane of semi-infinite interlayer-substituted herbertsmithite (for further details see Supplemental Information). The obtained spectral function *A*(*k, ω*) of the surface layer in chain termination is shown in Fig. 5, where the *k*-path is chosen perpendicular to the direction in which surface states propagate.

The hole-doped case clearly shows only one surface state of one spin species crossing the Fermi level (QAHE, see Fig. 5a), while the electron-doped case shows two surface states with opposite spin (QSHE, see Fig. 5b). The spectral function of the dual surface (triangles termination) has the same essential features (shown in the Supplemental Information). As we take into account realistic bandstructures, our spectral functions show additional structure away from the Fermi level compared to model calculations in next-neighbor approximation^21^,^22^.

## Discussion

In this work we have presented a new generally applicable strategy for creating materials where the quantum anomalous Hall effect and the quantum spin Hall effect can be realized at affordable energy and temperature scales, based on existing kagome lattice Mott insulators.

For the quantum anomalous Hall effect we showed that if the Fermi level is placed into the kagome flat band, the reconstructed bands are fully spin-polarized and show a topologically non-trivial gap at the Fermi level with conducting surface states of only one spin species. We demonstrated our proposal by considering the kagome Mott insulator herbertsmithite. Although the kagome flat band is only nearly flat in the real system, a quantum anomalous Hall state with Curie temperature well above 1000 K is established upon chemical substitution. The correlated nature of the Cu 3*d* orbitals plays an important role for the existence of fully spin-polarized bands with a gap to the empty minority bands. As we have been dealing with 3*d* electrons, the calculated spin-orbit induced band gap is of the order of 15–20 meV. Our scheme is applicable to 4*d* and 5*d* systems, where significantly larger spin-orbit band gaps are to be expected, while still preserving some correlation effects.

Electron doping of herbertsmithite up to the Dirac point yields, on the other hand, a topological insulator (QSHE). With the earlier prediction of superconductivity close to the Dirac point^12^, the Ga_*x*_Zn_1−*x*_ -herbertsmithite system might present an interesting platform for building a topological quantum computer by locally controlling the Ga:Zn ratio.

Synthesis of such doped kagome systems may be a challenge. However, our calculations show a robust stability of the structures and correctly predict, for instance, that the Cd-substituted herbertsmithite distorts, as has been observed experimentally^53^. This gives some reassurance about the predictive power and actual realization of the phenomena proposed in the present work. Nevertheless, chemical doping may not be the only route to achieve hole or electron doping in herbertsmithite. In recent years a few alternative techniques have been very successful in doping Mott insulators like deposition of alkali ions^54^ or gating the materials with ionic liquids^55^,^56^. For instance, it has recently become possible to tune the critical temperature of La_2_CuO_4+*x*_ thin films by gating the parent compound^57^. Following different doping routes may allow the realization of our predictions.

## Additional Information

**How to cite this article**: Guterding, D. *et al*. Prospect of quantum anomalous Hall and quantum spin Hall effect in doped kagome lattice Mott insulators. *Sci. Rep.* **6**, 25988; doi: 10.1038/srep25988 (2016).

## Supplementary Material

Supplementary Information

## Figures and Tables

**Figure 1:**
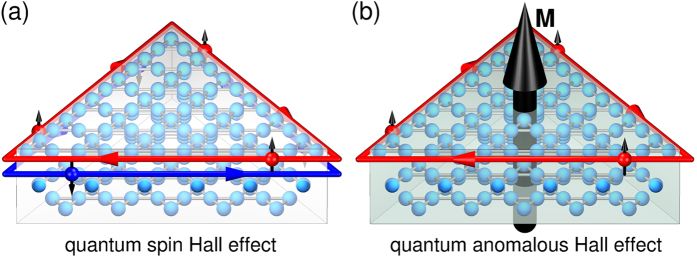
Predicted quantum Hall effects in doped herbertsmithite. (**a**) Quantum spin Hall effect (QSHE) with counterpropagating edge currents of opposite spin-polarization. (**b**) Quantum anomalous Hall effect (QAHE) with a single spin-polarized edge current due to intrinsic magnetization. In both cases the crystal is cut into triangular shape. The crystal structure shows the arrangement of copper atoms (light blue balls) in three shifted kagome planes as in herbertsmithite.
