# Supplementary material for: Prospect of quantum anomalous Hall and quantum spin Hall effect in doped kagome lattice Mott insulators
Source: Sci Rep. 2016 May 17;6:25988. doi: 10.1038/srep25988 (PMC4868970; doi:10.1038/srep25988)
Supplement: Supplementary Information [file srep25988-s1.pdf]

Supplemental Material: Prospect of quantum anomalous Hall and quantum spin Hall effect in doped kagome lattice Mott insulators

Daniel Guterding,* Harald O. Jeschke, and Roser Valentí
Institut für Theoretische Physik, Goethe-Universität Frankfurt, Max-von-Laue-Straße 1, 60438 Frankfurt am Main, Germany

I. FORMATION AND DOPING ENERGIES

Total energies were evaluated using *ab-initio* density functional theory (DFT) calculations within an all-electron full-potential local orbital (FPLO) [1] basis. For the exchange-correlation functional we employ the generalized gradient approximation (GGA) [2]. Total energies were extracted from calculations converged using 8^3 k-point grids.

Experimental and hypothetical crystal structures were fully relaxed using the projector augmented wave (PAW) method [3] implemented in GPAW [4] with a plane-wave cutoff of 1000 eV and the GGA exchange-correlation functional [2]. We optimized the stoichiometric structures of herbertsmithite-based compounds using 6^3 k-points (4^3 k-points for non-stoichiometric structures) until forces were below $10\,\mathrm{meV/Å}$.

Hypothetical crystal structures were prepared starting from the experimental crystal structure of herbertsmithite [5], substituting zinc (Zn) atoms between the copper kagome layers by monovalent A=Li, Na (hole-doping) and trivalent Al, Ga, In, Sc, Y (electron-doping). We refer to these compounds as A-herbertsmithite, $A\mathrm{Cu_3(OH)_6Cl_2}$ (case 1) and estimate their stability by comparison to clinoatacamite [$\mathrm{Cu_2(OH)_3Cl}$]. We directly compare energies of A-substituted materials, where the dopant sits in the interlayer site versus structures where it occupied a kagome site (case 2, see Fig. 3 in main paper). To analyze the phase stability, we also evaluate total energies of $3 \times 1 \times 1$ supercell structures, where either one of the A-sites is vacant [$A_{0.66}\mathrm{Cu_3(OH)_6Cl_2}$, case 3] or occupied by a Cu impurity [$A_{0.66}\mathrm{Cu_{3.33}(OH)_6Cl_2}$, case 4].

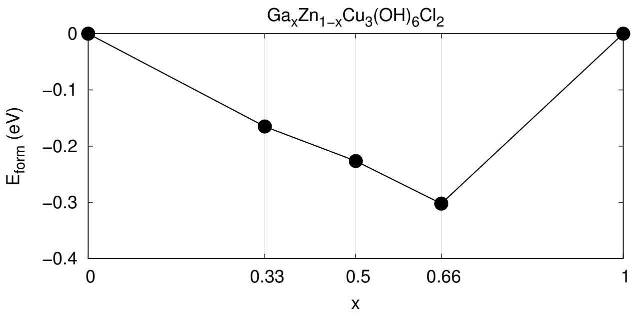

FIG. 1. Formation energies of the $\mathrm{Ga}_x\mathrm{Zn}_{1-x}\mathrm{Cu_3(OH)_6Cl_2}$ doping series. Lines are guides to the eye.

In order to determine whether the presence of excess copper in the synthesis process makes the formation of clinoatacamite favorable compared to $A\mathrm{Cu_3(OH)_6Cl_2}$, we compare the total energies of Cu in a copper crystal plus the energy of $A\mathrm{Cu_3(OH)_6Cl_2}$ to the total energy of A in a crystal of substance A plus the total energy of clinoatacamite. Proper normalization of total energies to formula units is taken into account. The calculated energy difference determines whether the formation of metallic patches of substance A is energetically favourable compared to forming A-substituted herbertsmithite. The formation energy for this case is defined as

$$E_{\mathrm{form}} = E_{\mathrm{Cu}} + E_{\mathrm{AH}} - (E_A + E_{\mathrm{C}})\,, \qquad (1)$$

where E_{A} refers to the pure crystalline metal A, E_{AH} to A-herbertsmithite and E_{C} to clinoatacamite. As all total energies are negative, a negative formation energy signals stability of the target compound $A\mathrm{Cu_3(OH)_6Cl_2}$. As a consistency check, we also prepared a structure with copper on the interlayer site so that the chemical formula is identical to clinoatacamite. For this structure, we find a positive formation energy. Therefore, hypothetical Cu-herbertsmithite is unstable with respect to the formation of clinoatacamite, as expected.

For the formation energy of impurity or vacancy structures, we use

$$E_{\mathrm{form}} = y \cdot E_{\mathrm{Cu}} + E_{\mathrm{AH}} - (x \cdot E_A + E_{\mathrm{AHM}})\,, \qquad (2)$$

where E_{AHM} is the total energy of the compound $A_{1-x}\mathrm{Cu}_{3+y}\mathrm{(OH)_6Cl_2}$.

Formation energies for modifications of herbertsmithite are shown in Table I. As all calculated energy differences are negative, formation of A-herbertsmithite is always favorable compared to formation of clinoatacamite. All proposed modifications are robust against formation of vacancies or copper impurities on the (interlayer) A-site.

The doping energies shown in Fig. 3 of the main paper are computed from energy differences between structures with dopant atoms occupying the interlayer sites and structures with dopant atoms on a kagome site (case 2). The values for the doping energies are given in Table I, together with ionic radii taken from Ref. [6].

Having established the stability of stoichiometric compounds, we investigate the solid solution of Zn and Ga

* guterding@itp.uni-frankfurt.de

TABLE I. Calculated formation and doping energies for substituted herbertsmithite. Energies are given in eV (electron volts) per formula unit of A-substituted herbertsmithite $ACu_3(OH)_6Cl_2$ (A = Li, Na, Zn, Cd, Sc, Y, Al, Ga, In). Negative values signal stability of the stoichiometric compound against the formation of clinoatacamite (case 1), doping into the kagome layer (case 2, see Fig. 3 in main paper), vacancies on the interlayer site (case 3) and impurities on the interlayer site (case 4). Ionic radii in coordination number 6 are taken from Ref. [6]. In this configuration Cu^{2+} has an ionic radius of 72 pm.

case	A-herbertsmithite, A =	Li^{1+}	Na^{1+}	Zn^{2+}	Cd^{2+}	Sc^{3+}	Y^{3+}	Al^{3+}	Ga^{3+}	In^{3+}
1	$ACu_3(OH)_6Cl_2$	-2.660	-2.517	-2.082	-1.461	-7.300	-7.144	-6.257	-2.939	-3.080
2	$ACu_3(OH)_6Cl_2$	-0.041	+0.257	-0.261	+0.128	-0.372	+0.043	-1.101	-0.736	-0.203
3	$A_{0.66}Cu_3(OH)_6Cl_2$	-0.847	-0.735	-0.694	n/a	-2.481	-2.398	-2.141	-1.176	-1.062
4	$A_{0.66}Cu_{3.33}(OH)_6Cl_2$	-1.497	-1.350	-1.321	n/a	-3.019	-2.878	-2.659	-1.711	-1.585
	ionic radius in pm	76	102	74	95	75	90	54	62	80

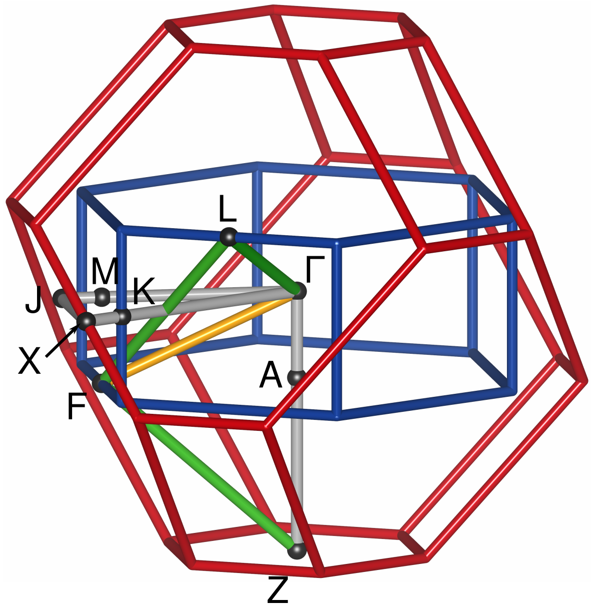

FIG. 2. Brillouin zone of herbertsmithite with high-symmetry points. The space group is $R\bar{3}m$. The path conventionally used for showing the bandstructure of herbertsmithite is indicated in grey. A path comprising the time-reversal invariant points is shown in green. The path on which surface states were calculated is shown in yellow.

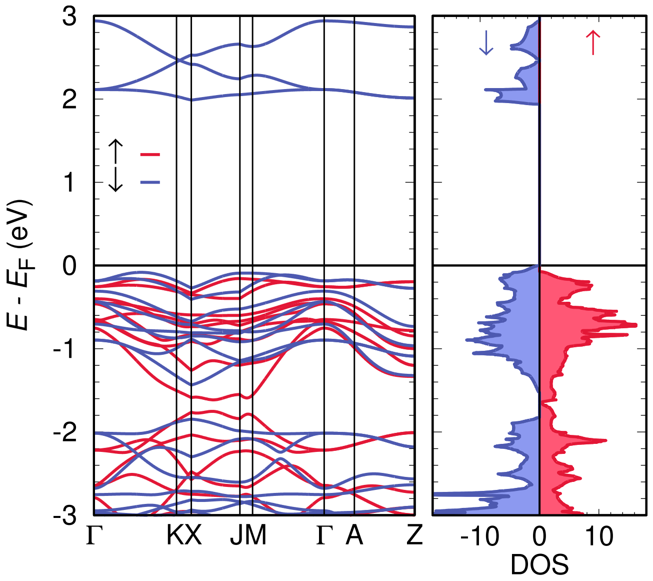

FIG. 3. Spin-polarized electronic bandstructure of herbertsmithite in ferromagnetic spin-configuration calculated from DFT+U. The interaction parameters on the Cu 3d shell are U = 6 eV and Hund's rule coupling J_H = 1 eV. A gap of about 2 eV is opened at the Fermi level due to electron-electron interactions.

dopants. To accomodate non-integer dopant concentrations, we use $2 \times 1 \times 1$ and $3 \times 1 \times 1$ super cells. The formation energy of the target compound having total energy E_T is defined as

$$E_{form} = E_T + x \cdot (E_{Zn} - E_{Ga}) - (1-x) \cdot E_{ZnH} - x \cdot E_{GaH}, \quad (3)$$

where E_{ZnH} refers to herbertsmithite and E_{GaH} to Gallium-substituted herbertsmithite. Note that as a reference energy here we use $(1-x) \cdot E_{ZnH} + x \cdot E_{GaH}$, which is the energy that one obtains if the solid solution were to dissociate spontaneously into herbertsmithite and Ga-substituted herbertsmithite. Therefore, formation energies are negative if the solid solution is stable against phase separation. Our results show that the solid solution $Ga_xZn_{1-x}Cu_3(OH)_6Cl_2$ should exist in a broad range of doping ratios (Fig. 1).

II. DETAILS ON PREPARATION OF HYPOTHETICAL CRYSTAL STRUCTURES

For all cases discussed in the previous section, crystal structures had to be prepared from a stoichiometric structure of herbertsmithite [5]. We started from the rhombohedral unit cell of space group $R\bar{3}m$, containing one formula unit. After reducing symmetry to space group $P1$, herbertsmithite allows for only one possibility to arrange dopant atoms or defects for all compositions investigated here, if the smallest possible supercell is considered.

Fig. 2 shows the Brillouin zone with high-symmetry points for materials based on herbertsmithite.

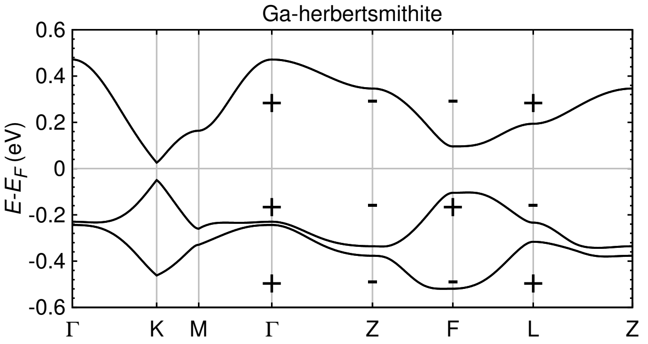

FIG. 4. Fully relativistic bandstructure of Ga-herbertsmithite. Plus and minus signs denote the parities of the three bands closest to the Fermi level at eight inversion-symmetric points (Γ, $3\times F$, $3\times L$, Z) in the Brillouin zone. In Ga-herbertsmithite the bandstructure is more three-dimensional than in Li-herbertsmithite, which leads to a substantial displacement of the Dirac point from the high-symmetry point K. In turn the apparent gap at K is exaggerated.

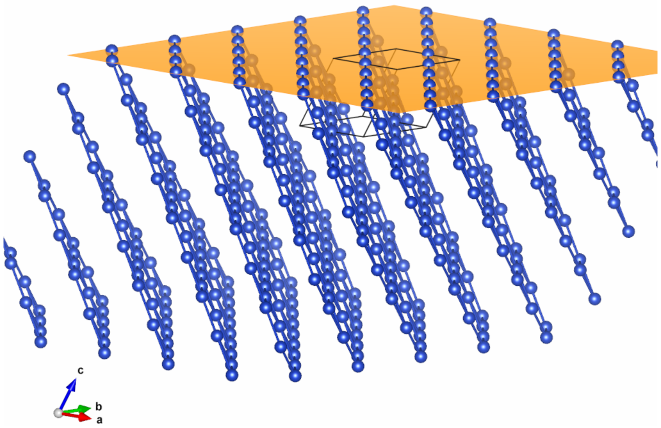

FIG. 5. (001) surface of herbertsmithite (shown as orange colored plane). Here, we used the predicted structure of Li-herbertsmithite. Ions other than Cu are omitted for clarity. The rhombohedral unit cell is shown in black. Note how all kagome layers are terminated with chains of copper ions at the surface.

III. BANDSTRUCTURE OF HERBERTSMITHITE IN FERROMAGNETIC SPIN-CONFIGURATION

Herbertsmithite is an antiferromagnetic Mott insulator with possible quantum spin liquid ground state. Although density functional theory cannot describe such a paramagnetic ground state with large fluctuating moments, a DFT+U calculation for the most simple ordered magnetic configuration (ferromagnetic) already reproduces the insulating ground state of herbertsmithite with a large band gap of about 2 eV (see Fig. 3).

IV. BANDSTRUCTURE AND TOPOLOGICAL PROPERTIES OF ELECTRON-DOPED HERBERTSMITHITE

We also analysed the bandstructure and topological properties of electron-doped herbertsmithite. As an example, in Fig. 4 we show the fully relativistic non-spin-polarized electronic bandstructure of Ga-herbertsmithite, where the parities of the three (dominantly Cu $d_{x^2-y^2}$) bands closest to the Fermi level are indicated. Ga-herbertsmithite is more three-dimensional than Li-herbertsmithite. Therefore, the Dirac point is displace from K, which exaggerates the gap at K. At the Dirac point the gap magnitude is the same as in Li-herbertsmithite.

The paritiy eigenvalues of the bands closest to the Fermi level are the same as in all other compounds derived from herbertsmithite that we predict to be stable. That means, topological numbers of the bands below the Dirac point are $\nu_0;(\nu_1,\nu_2,\nu_3) = 0;(111)$.

V. FORMALISM FOR COMPUTING SURFACE STATES

We use the Green's function method for the semi-infinite system [7–9] to calculate the states on the (001) surface of interlayer substituted herbertsmithite. In this scheme, the crystal must be partitioned into so-called principal layers parallel to the surface of interest. The Hamiltonian within the principal layer is denoted as H_0, while the coupling between neighboring principal layers is denoted as C. Note that only hoppings with components in direction either negative or positive perpendicular to the surface must be included into C. The corresponding couplings in the reverse direction are taken into account in the formalism by the adjoint matrix $C^\dagger$.

The surface Green's function of a system consisting of N principal layers can be written as

$$G_{ij}^{(N)}(\omega) = \left[\left(\omega - H_0 - CG^{(N-1)}C^\dagger\right)^{-1}\right]_{ij}. \quad (4)$$

The initial condition is

$$G_{ij}^{(1)}(\omega) = (\omega - H_0)_{ij}^{-1}. \quad (5)$$

Indices i,j denote combined site, orbital and spin variables. The Green's function of the dual surface can be calculated by iterating

$$G_{ij}^{(N)}(\omega) = \left[\left(\omega - H_0 - C^\dagger G^{(N-1)}C\right)^{-1}\right]_{ij}. \quad (6)$$

As we use Green's functions on the real-frequency axis, an artificial imaginary part must be added to ensure numerical convergence. To this end, we transform $\omega \to \omega + i\eta$ with $\eta = 10^{-5}$ eV in the equations above.

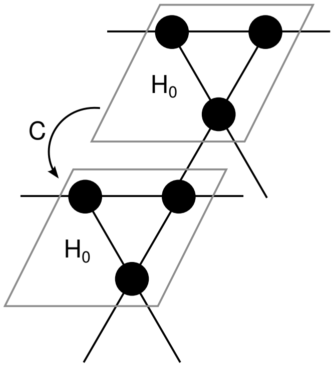

FIG. 6. Schematic partitioning of the tight-binding Hamiltonian into layers parallel to the surface. The principal layer is described by Hamiltonian H_0, while C contains hopping elements connecting two adjacent principal layers. In the real herbertsmithite system longer range hoppings are included and two kagome layers belong to one unit cell. For comparison, see Fig. 5.

Although the method in principle allows for the treatment of semi-infinite systems, the numerical iteration of Eq. (4) or (6) has to be stopped with a finite number of layers. We use $N = 10^5$.

VI. (001) SURFACE OF HERBERTSMITHITE

In the main paper we presented topological invariants $\nu_0; (\nu_1, \nu_2, \nu_3) = 0; (111)$ for the herbertsmithite system. According to Ref. [10], a material with those topological invariants displays conducting states on a (001) surface. The orientation of this lattice plane refers to the unit cell in rhombohedral setting of space group $R\bar{3}m$. The (001) surface of Li-herbertsmithite is shown in Fig. 5. Note how the Cu ions at the surface are arranged in chains, with all kagome layers terminated equivalently.

The unit cell of (substituted) herbertsmithite contains in total three Cu ions. These three ions comprise the minimal model of the herbertsmithite material. In the (001) surface geometry we use, two of the ions are at the surface and one is below the surface. The minimal principal layer therefore comprises Cu ions at different heights within the unit cell. This complicates the partitioning of hopping elements into matrices H_0 and C needed for the Green's function method. The situation is depicted in Fig. 6.

As mentioned in the main paper, and obvious from Fig. 6, the semi-infinite system can be built up either in positive or negative direction perpendicular to the surface. In the kagome lattice this results in an edge termination with either chains or triangles. The case of chains is presented in the main paper. For the dual surface (triangles termination) one obtains a spectral function with equivalent features (see Fig. 7) from Eq. (6).

Both in the main paper and in the supplement we show the spectral function $A(k, \omega)$ only in positive k-direction

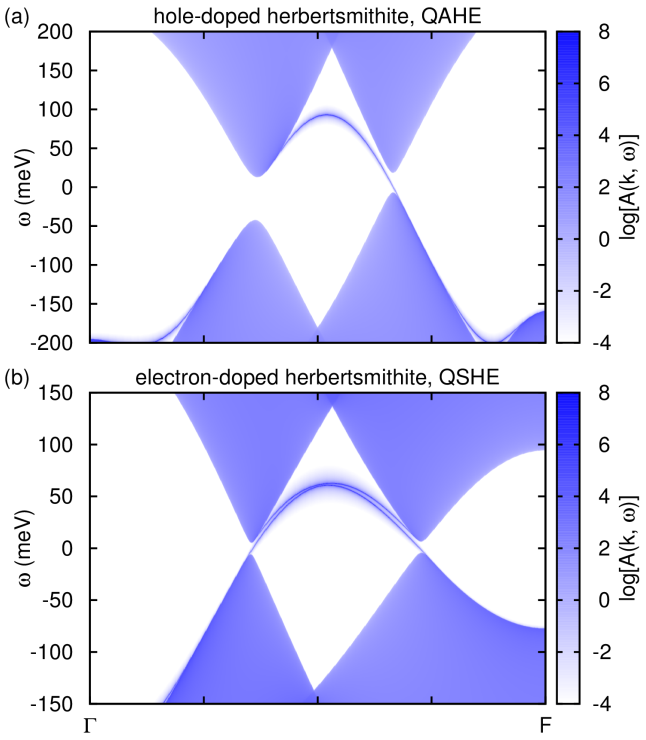

FIG. 7. Calculated states on the dual surface of substituted herbertsmithite. Spectral function on the (001) surface of (a) Li-herbertsmithite (hole-doped) and (b) Ga-herbertsmithite (electron-doped) calculated using Green's functions for the semi-infinite system. The kagome layers are terminated with triangles.

within the k_x-k_y-plane. As can bee seen from Fig. 5, the surface unit cell of (001) herbertsmithite contains two chains of Cu ions. The surface is mirror symmetric with respect to the k_x and the k_y-direction. Therefore, the symmetry equivalent edge states of the second kagome layer appear in negative k-direction.

VII. DETERMINATION OF HEISENBERG HAMILTONIAN PARAMETERS

Based on the theoretically predicted structures of Li-herbertsmithite (see Table II, Fig. 8a), we use density functional theory calculations to determine the most important couplings of the Heisenberg Hamiltonian. We use the all electron full potential local orbital (FPLO) code [1] with a generalized gradient approximation [2] exchange and correlation functional and correct for the strong correlations on the Cu^{2+} $3d$ orbitals using GGA+U [11].

The results for $LiCu_3(OH)_6Cl_2$ are given in Table III. The calculation was performed with a $\sqrt{2} \times \sqrt{2} \times 2$ supercell of the rhombohedral primitive cell of Li-herbertsmithite. With $P1$ symmetry, this cell contains four formula units, *i.e.* twelve inequivalent Cu sites. This

TABLE II. Predicted structural parameters for Li-herbertsmithite [$LiCu_3(OH)_6Cl_2$]. ($R\bar{3}m$ space group, $a = 6.96433$ Å, $c = 13.78483$ Å, $Z = 3$)

Atom	Site	x	y	z
Cu	$9d$	1/2	0	1/2
Li	$3a$	0	0	0
O	$18h$	-0.208833	0.208833	0.447033
H	$18h$	-0.131973	0.131973	0.420633
Cl	$6c$	0	0	0.328170

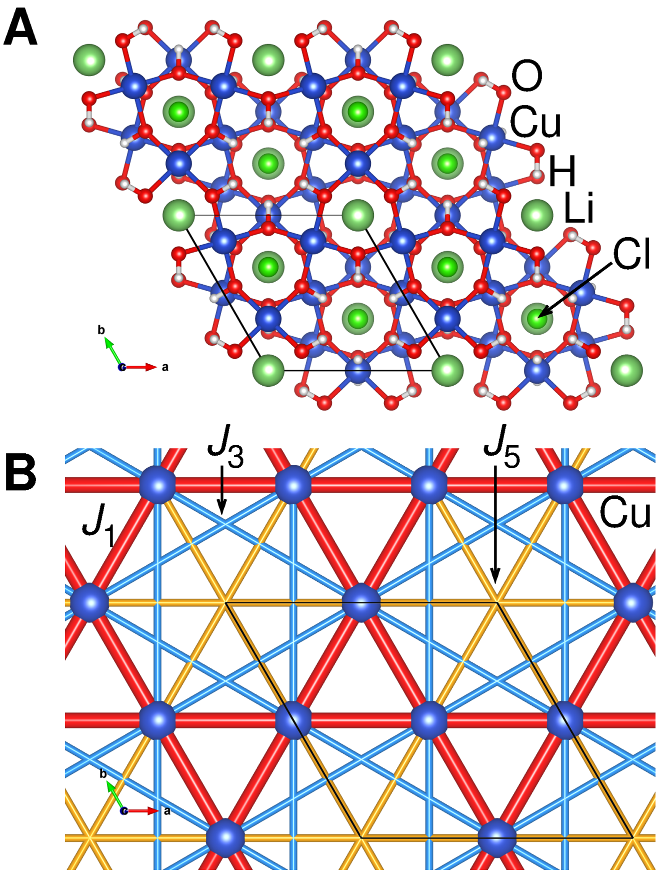

FIG. 8. Predicted crystal structure and exchange paths in Li-herbertsmithite. (a) View of the Li-herbertsmithite structure along c direction (in hexagonal setting of the $R\bar{3}m$ space group). (b) Detail of the $LiCu_3(OH)_6Cl_2$ unit cell with the first three inplane exchange paths between Cu^{2+} ions. Other ions are omitted for clarity.

TABLE III. Calculated exchange couplings for predicted Li-herbertsmithite, $LiCu_3(OH)_6Cl_2$. A GGA+U functional with $J_H = 1$ eV and the two listed U values was used in combination with fully localized limit double counting correction.

U (eV)	J_1 (K)	J_2 (K)	J_3 (K)	J_5 (K)	J_6 (K)
6.0	-544(29)	7(15)	39(24)	-50(13)	2(8)
8.0	-332(32)	-13(16)	-11(27)	-75(20)	1(8)

allows for 171 out of 4096 unique spin configurations, and the data in Table III are based on fits to total energies for about 30 of these configurations. The exchange couplings are given assuming $S = 1/2$. However, due to the changed filling of the Cu bands, the moments in the calculation are reduced from 1 μ_B to, on average, about 0.67 μ_B. The relevant exchange paths are visualized in Fig. 8b. Based on previous work [12], we consider the GGA+U functional with $U = 6$ eV, $J_H = 1$ eV most relevant for Cu in the square planar environment as realized in herbertsmithite type crystals.

VIII. ESTIMATE FOR THE CURIE TEMPERATURE

We estimate the Curie temperature T_C from a simple Weiss mean-field formula [13]. For 3d transition metals, in this approximation the Curie temperature is given by Eq. (7), where z_i is the coordination number and J_i are the exchange couplings in Kelvin ($U = 6$ eV), taken from Table III.

$$T_C = -\frac{2}{3}S(S+1)\sum_i z_i J_i \tag{7}$$

We obtain $T_C = 1160$ K, where we took into account $i = \{1, 3, 5\}$ with $z_i = \{4, 4, 6\}$. Instead of the spin-1/2 model used here, it is also conceivable to parametrize the Heisenberg model with DFT effective moments for S, which would result in a higher value for T_C.

[1] K. Koepernik and H. Eschrig, *Full-potential nonorthogonal local-orbital minimum-basis band-structure scheme*, Phys. Rev. B **59**, 1743 (1999); http://www.FPLO.de

[2] J. P. Perdew, K. Burke, and M. Ernzerhof, *Generalized Gradient Approximation Made Simple*, Phys. Rev. Lett. **77**, 3865 (1996).

[3] P. E. Blöchl, *Projector augmented-wave method*, Phys. Rev. B **50**, 17953 (1994).

[4] J. Enkovaara, C. Rostgaard, J. J. Mortensen *et al.*, *Electronic structure calculations with GPAW: a real-space implementation of the projector augmented-wave method*, J. Phys.: Condens. Matter **22**, 253202 (2010); https://wiki.fysik.dtu.dk/gpaw

[5] M. P. Shores, E. A. Nytko, B. M. Bartlett, and D. G. Nocera, *A Structurally Perfect S=1/2 Kagomé Antiferromagnet*, J. Am. Chem. Soc. **127**, 13462 (2005).

[6] R. D. Shannon, *Revised Effective Ionic Radii and Systematic Studies of Interatomic Dinstances in Halides and Chalcogenides*, Acta Cryst. A **32**, 751 (1976).

[7] M. P. López Sancho, J. M. López Sancho, and J. Rubio, *Quick iterative scheme for the calculation of transfer matrices: application to Mo(100)*, J. Phys. F: Met. Phys. **14**, 1205 (1984).

[8] M. P. López Sancho, J. M. López Sancho, and J. Rubio, *Highly convergent schemes for the calculation of bulk and surface Green functions*, J. Phys. F: Met. Phys. **15**, 851 (1985).

[9] X. Dai, T. L. Hughes, X.-L. Qi, Z. Fang, and S.-C. Zhang, *Helical edge and surface states in HgTe quantum wells and bulk insulators*, Phys. Rev. B **77**, 125319 (2008).

[10] L. Fu and C. L. Kane, *Topological insulators with inversion symmetry*, Phys. Rev. B **76**, 045302 (2007).

[11] A. I. Liechtenstein, V. I. Anisimov, and J. Zaanen, *Density-functional theory and strong interactions: Orbital ordering in Mott-Hubbard insulators*, Phys. Rev. B **52**, R5467(R) (1995).

[12] H. O. Jeschke, F. Salvat-Pujol, and R. Valentí, *First-principles determination of Heisenberg Hamiltonian parameters for the spin-1/2 kagome antiferromagnet* $ZnCu_3(OH)_6Cl_2$, Phys. Rev. B **88**, 075106 (2013).

[13] S. Blundell, *Magnetism in Condensed Matter* (Oxford University Press, Oxford, 2010).
